# Mechanisms of Flavivirus Cross-Protection against Yellow Fever in a Mouse Model

**DOI:** 10.3390/v16060836

**Published:** 2024-05-24

**Authors:** Divya P. Shinde, Jordyn Walker, Rachel A. Reyna, Dionna Scharton, Brooke Mitchell, Ennid Dulaney, Srinivisa Reddy Bonam, Haitao Hu, Jessica A. Plante, Kenneth S. Plante, Scott C. Weaver

**Affiliations:** 1 Department of Microbiology and Immunology, University of Texas Medical Branch, Galveston, TX 77555, USA; dimircha@utmb.edu (D.P.S.); jolwalke@utmb.edu (J.W.); rasattle@utmb.edu (R.A.R.); dionnascharton@gmail.com (D.S.); bmmitche@utmb.edu (B.M.); earosale@utmb.edu (E.D.); srbonam@utmb.edu (S.R.B.); haihu@utmb.edu (H.H.); japlante@utmb.edu (J.A.P.); 2 World Reference Center for Emerging Viruses and Arboviruses, University of Texas Medical Branch, Galveston, TX 77555, USA; 3 Institute for Human Infections and Immunity, University of Texas Medical Branch, Galveston, TX 77555, USA

**Keywords:** mosquito-borne viruses, cross-protection, mouse model, dengue, Zika, yellow fever virus

## Abstract

The complete lack of yellow fever virus (YFV) in Asia, and the lack of urban YFV transmission in South America, despite the abundance of the peridomestic mosquito vector *Aedes* (*Stegomyia.*) *aegypti* is an enigma. An immunologically naïve population of over 2 billion resides in Asia, with most regions infested with the urban YF vector. One hypothesis for the lack of Asian YF, and absence of urban YF in the Americas for over 80 years, is that prior immunity to related flaviviruses like dengue (DENV) or Zika virus (ZIKV) modulates YFV infection and transmission dynamics. Here we utilized an interferon α/β receptor knock-out mouse model to determine the role of pre-existing dengue-2 (DENV-2) and Zika virus (ZIKV) immunity in YF virus infection, and to determine mechanisms of cross-protection. We utilized African and Brazilian YF strains and found that DENV-2 and ZIKV immunity significantly suppresses YFV viremia in mice, but may or may not protect relative to disease outcomes. Cross-protection appears to be mediated mainly by humoral immune responses. These studies underscore the importance of re-assessing the risks associated with YF outbreak while accounting for prior immunity from flaviviruses that are endemic.

## 1. Introduction

Mosquito-transmitted viruses produce a substantial global health burden, causing hundreds of millions of infections each year, with multiple viruses often co-existing in various regions worldwide [1]. Dengue viruses (DENV 1-4) are responsible for an estimated 390 million infections, particularly in hyperendemic regions like Asia and Latin America, where multiple serotypes circulate concurrently [2]. The emergence of Zika virus (ZIKV) in the Americas in 2014 led to a major outbreak [3,4]. Additionally, several other mosquito-borne arboviruses, such as chikungunya, Japanese encephalitis, West Nile, St. Louis encephalitis, and Venezuelan equine encephalitis viruses, sporadically cause outbreaks in various parts of the world [5,6,7,8,9,10]. It is therefore important to consider not only direct the transmission dynamics of viral determinants but the context in which transmission takes place, especially regarding pre-existing immunity to locally circulating, antigenically related viruses.

Yellow fever virus (YFV), a flavivirus [11] transmitted by mosquitoes, is typically maintained in an enzootic cycle between arboreal mosquitoes and non-human primates [12,13,14]. Human intrusion into forested areas can lead to spillover infections, and sometimes to the establishment of a human-amplified urban cycle [15,16]. *Aedes* (*Stegomyia*) *aegypti*, the primary mosquito vector for urban YFV transmission, also transmits DENV and ZIKV throughout the tropics [1]. This anthropophilic species, originally from Africa, has now spread across tropical and sub-tropical regions worldwide, infesting much of Africa, Southeast Asia, and the Americas [17]. The prevalence of these mosquitoes makes human populations in these regions at high risk for diseases like dengue, Zika, chikungunya, and yellow fever. While DENV is hyperendemic in these areas, reports of ZIKV occurrence are also widespread, coinciding with the vector distribution. Conversely, there are major discrepancies between the distribution of *Ae. aegypti* and that of YF. Historically, YFV emerged in Africa and caused devastating outbreaks after importation to the New World, resulting in major outbreaks accompanied by fatalities and economic losses. However, YFV has never been found circulating in Asia, where over 2 billion immunologically naïve people reside in areas infested with *Ae. aegypti* [17,18,19,20].

Thus, an intriguing question has persisted for over a century: despite the conducive environment and abundance of the mosquito vector, why is there no occurrence of YFV transmission in Asia? Moreover, during the Brazilian outbreak of YF from 2016–2019, although thousands of severe human and non-human primate infections and numerous fatalities were documented, the virus apparently failed to establish a human-amplified transmission cycle, persisting only in sylvatic mosquitoes, with spillover to humans [21,22,23,24]. Thus, the enigma remains: what factors have acted as barriers preventing the urban emergence of YFV in South America for decades and prompting the complete historic absence of YFV in Asia? Various theories have been postulated to explain this, including geographical distance between YFV’s African origins and Asia, the potential inability of Asian *Ae. aegypti* to transmit YFV, and flavivirus-cross-reactive herd immunity, particularly from DENV [18,19,20,25,26]. DENV is hyper-endemic in both Asia and South America, and autochthonous ZIKV transmission has been reported in Asia since 1952, with estimates of its introduction from Africa dating back many decades [27,28]. Additionally, a major ZIKV outbreak in the Americas in 2015 preceded the YF outbreak in Brazil, resulting in over 50% of the population developing immunity to ZIKV prior to the YF epidemic [4,29,30]. These factors prompted us to inquire if DENV or ZIKV immunity modulates YFV infection.

In our previous studies with non-human primates and live-feeding mosquitoes, we showed that prior DENV and ZIKV immunity lowers YFV (Brazilian strain) viremia, further inhibiting mosquito infectivity and transmission potential [31]. Here, we expanded on this hypothesis, seeking to determine the impact of pre-existing immunity to DENV and ZIKV on African and South American YFV infection in a mouse model, and investigate the role of adaptive immune responses. Our findings reveal that (1) prior immunity significantly diminishes YFV viremia, with the effect varying depending on the YFV strain, (2) pre-existing immunity to DENV or ZIKV demonstrates a variable influence on YF disease outcomes, and (3) humoral DENV/ZIKV immunity appears to play a greater role than does cellular immunity in suppressing YFV viremia in mice.

## 2. Materials and Methods

**In vivo study design**

For morbidity and viremia studies, seven-to-eight-week-old A129 (*Ifnar*^−/−^) male mice (n = 5 per group) were sham-infected or infected, primarily with DENV-2 NGC (endemic strain), DENV-2 P81407 (sylvatic strain, hereafter referred to as DENV-2 P8), or ZIKV PRVABC59 (epidemic strain, hereafter referred as ZIKV PR) via a subcutaneous route on the scruff at a dose of 1 × 10^3^ FFU. Mice were allowed to build immunity for 7–8 weeks prior to secondary intraperitoneal (IP) inoculation with YFV BR (Brazilian strain isolated from an NHP in 2017 during an outbreak) or YFV Asibi (prototypic African strain originally isolated from a Ghanian man in 1937) at dose of 1 × 10^5^ FFU. Mice were observed daily for clinical signs and weighed regularly for 14 days post-infection (DPI). Prior to the YFV challenge, blood was collected via the retro-orbital route, and the separated sera were used to quantify neutralizing antibodies against respective homologous viruses (DENV-2 NGC, DENV-2 P8, or ZIKV PR) to ensure successful infection and seroconversion. Similarly, viremia levels were measured from blood collected at 2 DPI, which represents the peak viremia timepoint.

For pathogenesis studies, the primary and secondary infections in mice (n = 10 per group) were identical to those in the previous study. Upon YFV challenge, five mice from each group were sacrificed at 2 DPI and 4 DPI. At each timepoint, liver, kidney, and spleen were collected to measure viral load. Sera from blood collected at 4 DPI were analyzed using a Preventative Care Profile Plus rotor on a VetScan VS2 Chemistry Analyzer (Abaxis, Union City, CA, USA).

All experiments were performed in full compliance with the guidelines established by the Animal Welfare Act for the housing and care of laboratory animals and conducted as laid out in the University of Texas Medical Branch Institutional Animal Care and Use Committee (UTMB-IACUC) approved protocol (protocol #1708051B, approved 28 August 2017). The progenitors of the A129 mice were originally purchased from Marshall BioResources, and the current subjects were bred in-house at UTMB.

**Cells**

Vero E6 (African green monkey kidney epithelial) cells were grown in a modified essential medium (MEM, Gibco, Grand Island, NY, USA) supplemented with 10% heat-inactivated fetal bovine serum (FBS, Atlanta Biologics, Flowery Branch, GA, USA), 1% penicillin-streptomycin solution (PenStrep, Gibco, Grand Island, NY, USA), 1% sodium bicarbonate (Gibco, Grand Island, NY, USA), and 1% glutamax (Gibco, Grand Island, NY, USA). C6/36 cells were grown in MEM and L-15 media at a ratio of 1:1, supplemented with 10% FBS, 1% PennStrep, 1% glutamax, 1% sodium bicarbonate, 10% tryptose phosphate broth solution (TPB, Sigma, St Louis, MO, USA), and 2% non-essential amino acid solution (Sigma, St Louis, MO, USA). Vero E6 and C6/36 cells were maintained at 37 °C and 28 °C, respectively, with 5% CO_2_. 

**Viruses**

All virus stocks were acquired from the World Reference Center for Emerging Viruses and Arboviruses at UTMB. The YFV BR17 stock was originally acquired from Dr. Betânia Drumond, Universidade Federal de Minas Gerais, Brazil. The virus was isolated from an infected *Alouatta guariba* in 2017 in Minas Gerais during the YF epidemic. The virus was passaged three times in C6/36 cells (*Ae. albopictus* cell line) and once in Vero E6 cells to generate a low-passage contemporary YFV BR17 stock which was utilized for the challenge. The YFV Asibi stock was originally acquired from human sera, passaged six times in monkeys, four times in C6/36 cells, and once in Vero E6 cells. DENV-2 NGC originally isolated from infected human serum from New Guinea was passaged once in monkey, and six times in C6/36 cells. DENV-2 P8 was originally isolated from a sentinel macaque in Malaysia and passaged three times in C6/36 cells. ZIKV PR was originally isolated from human serum in Puerto Rico during an epidemic and passaged five times in Vero E6 cells.

**Focus-forming assay**

Focus-forming assays were performed as previously described [31]. Briefly, Vero E6 cells were seeded in 12-well plates and cultured at 37 °C with 5% CO_2_ for approximately 24 h until 95% confluency. Serum samples collected at 2 DPI were used to measure viremia. To measure viral titers in organs, tissues were homogenized at a frequency of 26/s for 1 min in a TissueLyser II (Qiagen, Germantown, MD, USA) and centrifuged at 16,500× *g* for 5 min. Samples were serially diluted with Vero maintenance media (MEM, 2% FBS, 1% Penn-Strep, 1% Glutamax, and 1% sodium bicarbonate), transferred to the pre-seeded Vero E6 cells in 12-well plates, and incubated at 37 °C with 5% CO_2_ for 1 h. After incubation, 1 mL of overlay media (Opti-MEM (Gibco, Grand Island, NY, USA) supplemented with 1% carboxymethyl cellulose (Sigma Aldrich, St. Louis, MO, USA), 2% FBS, and 1% Penn-Strep was added to each well. After a 3-day (for ZIKV PR), 4-day (for YFV Asibi) or 5-day (for YFV BR and DENV-2) incubation, formalin was added to fix the infected plates. Plates were washed thrice with 1× DPBS (Gibco, Grand Island, NY, USA), followed by blocking with 5% (*w*/*v*) non-fat dry milk (Apex Chemicals and Reagents, Houston, TX, USA) in 1× DPBS for 30 min on a plate rocker. After blocking, anti-YFV primary antibody (mouse immune ascitic fluid acquired from WRCEVA) diluted at 1:1000 in blocking buffer was added and allowed to bind overnight. On the following day, the plates were washed (3× with 1× DPBS); this was followed by the addition of the secondary antibody (affinity purified antibody peroxidase labeled goat anti-mouse IgG, SeraCare, Milford, MA, USA) diluted at 1:2000 in blocking buffer and incubated on the plate rocker for 1 h. Following a final three washes, plates were stained using KPL TrueBlue Peroxidase Substrate (SeraCare, Milford, MA, USA) and foci were counted to determine the final infectious viral load.

**Focus-reduction neutralization test (FRNT)**

To determine the neutralizing capacity of antibodies in sera, FRNTs were conducted on baseline samples (prior to YFV infection), as previously described [31]. Samples from mice that were infected with DENV-2 NGC, DENV-2 P8-1407, or ZIKV PR were tested against a homologous virus. Briefly, sera were serially diluted two-fold in Vero maintenance media and mixed with 80 FFU of the respective virus. The serum–virus mixture was incubated at 37 °C with 5% CO2 for 1 h followed by infection of Vero E6 cells on the 12-well plates. Samples tested against DENV-2 were fixed on day 5, and those tested against ZIKV were fixed on day 3. Plates were stained, as described above, with the respective primary antibodies. The neutralizing titer was represented as the reciprocal of the highest dilution of serum that inhibited 50% of foci (FRNT_50_). Samples that scored below the limit of detection (<1:40) were considered seronegative.

**CD4^+^ T cell depletion**

The contribution of CD4^+^ T cells in sequential DENV-2/ZIKV and YFV infection was elucidated by depletion studies. Seven-to-eight-week-old A129 male mice were subcutaneously inoculated with either PBS, DENV-2 NGC, DENV-2 P8, or ZIKV PR at a dose of 1 × 10^3^ FFU and allowed to build immunity for 7–8 weeks, followed by an IP inoculation of PBS, YFV BR, or YFV Asibi. On days 3 and 1 prior to challenge with YFV BR or YFV Asibi, each mouse was injected intraperitoneally with 100 µg of anti-CD4^+^ antibody (clone GK1.5) (BioXcell, Lebanon, NH, USA). Control mice were injected with IgG2β isotype control (BioXcell, Lebanon, NH, USA). CD4^+^ T cell-depleted, YFV-infected mice were observed for morbidity up to 14 DPI, and blood was collected to determine viremia at 2 DPI.

Dose and injection timepoints for the anti-CD4 antibody were optimized in age and gender-matched mice to ensure that CD4^+^ T cells were depleted at the infection timepoint and stayed depleted beyond the study timepoint. Briefly, blood was collected via the retro-orbital route on day 3 and day 21 post-injection and stained for CD4^+^ and CD8^+^ T cells using anti-CD4 clone R4.5 (PE-cyanine) and anti-CD8 clone 5H10 (FITC). Successful CD4^+^ depletion was confirmed using flow cytometry, while CD8^+^ T cells were intact (Supplementary Figure S1). 

**Adoptive transfer of sera or CD8^+^ T cells**

To determine if CD8^+^ T cells contributed to the suppression of YFV viremia in DENV-2/ZIKV-immune mice, age- and gender-matched donor A129 mice were either sham-infected or DENV-2 NGC-, DENV-2 P8-, or ZIKV PR-infected. The dose and route were identical to those in previous groups. After 8 weeks, blood was collected to obtain sera. Spleens collected from each group were pooled in a single tube containing complete media (RPMI + 10% FBS + 1% Penn-Strep). Pooled spleens were manually dissociated in a Petri dish, with media used to isolate splenocytes. Cells were centrifuged at 1100× *g* for 5 min, the supernatant was discarded, and the pellets were resuspended in media. After three washes, splenocytes were resuspended in MACS buffer (MACS BSA Stock solution diluted at 1:20 with autoMACS rinsing solution). CD8^+^ T cells were isolated from splenocytes using a Miltenyi CD8a^+^ T cell isolation kit for mouse (Gaithersburg, MD, USA). Purity was confirmed by flow cytometry by staining with anti-CD3 clone 17A2 APC (Cat#100235), anti-CD4 clone GK1.5 FITC (Cat#100405), and anti-CD8 antibodies clone 53.6.7 PerCP at dilution of 1:40, and all samples were found to be >95% pure (Supplementary Figures S2–S5). Viable cells were counted using Trypan Blue and diluted in such a way that each mouse received one million CD8^+^ T cells.

For passive transfer of sera, pooled sera from each donor group were measured for homologous neutralizing antibodies. For the first study, 100 µL of sera from the respective groups was transferred to each recipient mouse, one day prior to YFV challenge. Morbidity and viremia measurements were identical to those of other studies. For the second study, to ensure that recipient mice received sufficient antibody, we increased the volume of sera transfer to 150 uL and incorporated a positive control group that received comparable levels of sera from YFV Asibi-infected mice. The remaining elements of the study design were the same.

**Statistical analyses**

Viremia and viral burden in organs were represented as log_10_ transformed values and analyzed by one-way ANOVA using Tukey’s multiple comparison test. Each group was compared with the flavivirus-naïve YFV-infected group. Weight changes were analyzed by two-way repeated measures ANOVA with Dunnet’s multiple comparison test. The *p*-values are as follows: ns: *p* > 0.05; * *p* < 0.05, ** *p* < 0.01, *** *p* < 0.001, and **** *p* < 0.0001. All analyses were performed using GraphPad Prism v.10.

## 3. Results

### 3.1. Prior Dengue-2 and Zika Virus Immunity Generally Suppresses Yellow Fever Viremia and Viral Loads in A129 Mice

**Figure 1:**
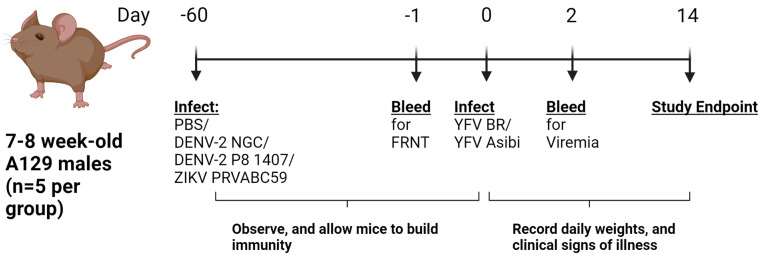
**Study Design**. Seven-to-eight-week-old A129 males were first inoculated with PBS or DENV-2 NGC, DENV-2 P8, or ZIKV PR, and allowed to develop immunity for 7–8 weeks. Mice were challenged with 10^5^ FFU YFV BR or Asibi and observed for morbidity and mortality up to 14 DPI. Sera at day −1 and day 2 were collected to measure neutralizing antibodies and viremia, respectively.

Male, 7–8-week-old A129 *Ifnar*^−/−^ mice were inoculated with PBS (n = 10) or 10^3^ focus-forming units (FFU) of either DENV-2 NGC, DENV-2 P8, or ZIKV PRV (n = 5 per infection group) via the subcutaneous route. Infected animals were allowed to develop immunity for 7–8 weeks before challenge with YFV BR or Asibi and compared with a sham-infected mock group (n = 5). Animals were weighed and monitored for clinical signs, and blood was collected at 2 DPI, which is the peak viremia timepoint, and this was used to measure YFV viremia levels (Figure 1).

To determine whether prior flavivirus immunity would reduce YFV load in organs, A129 males (n = 10 per group) were primarily and secondarily infected as previously described. Half of each cohort (n = 5) were serially sacrificed at 2 and 4 DPI. Liver, kidney, and spleen were collected without perfusion, to determine viral titers.

**Figure 2:**
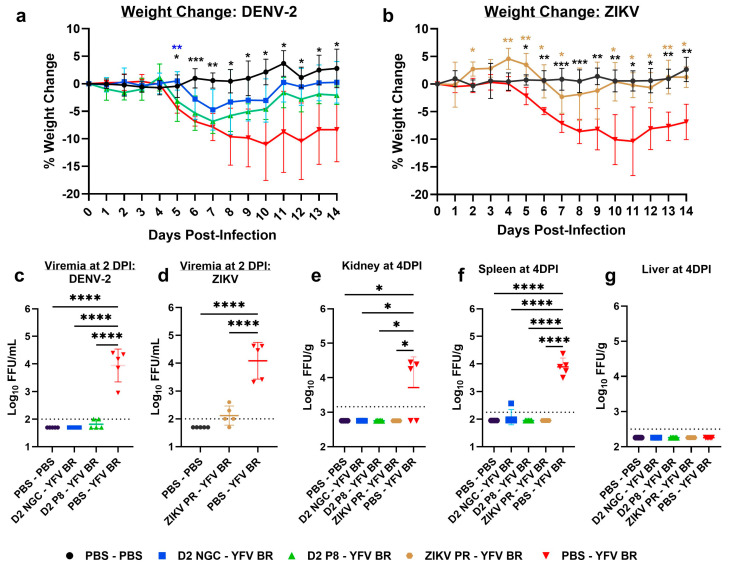
Percentage change in weight, and viremia and viral loads, in flavivirus-naïve and flavivirus-immune mice, post-YFV BR challenge. Seven-to-eight-week-old A129 male mice were first inoculated with PBS or inoculated with DENV-2 NGC (blue), DENV-2 P8 (green), or ZIKV PR (light brown), and allowed to develop immunity for 7–8 weeks. Mice were then challenged with 10^5^ FFU YFV BR and observed for morbidity and mortality, and viremia was measured at 2 DPI. The figure shows the percentage weight change in DENV-2 NGC-, DENV-2 P8- (**a**), and ZIKV-immune (**b**) mice, compared to flavivirus-naïve mice, post-YFV BR infection, respectively. Viremia levels are shown at 2 DPI in DENV-2 NGC-, DENV-2 P8- (**c**), and ZIKV- immune (**d**) mice post-YFV BR infection, respectively. In a separate study, PBS or YFV BR-challenged mice were sacrificed to collect kidney (**e**), spleen (**f**), and liver (**g**) at 4 DPI to determine viral loads. For weight-change graphs, symbols represent the mean, and error bars represent the standard deviation. For viral load graphs, symbols represent individual subjects, midlines represent the mean, and error bars represent the standard deviation. Samples with no detectable YFV are represented as half the lower limit of detection. Weight change is compared to the PBS-YFV BR group by two-way ANOVA and repeated measurements by Dunnet’s multiple comparisons test. Log_10_ transformed viremia levels are compared to PBS-YFV BR by one-way ANOVA with Tukey’s multiple comparison test. The *p*-values are as follows: * *p* < 0.05, ** *p* < 0.01, *** *p* < 0.001, and **** *p* < 0.0001.. Dotted line represents the limit of detection, and value below is represented as ½ of limit of detection.

Upon YFV BR challenge, all infected groups lost weight compared to the PBS mock group. However, the DENV-2 NGC, DENV-2 P8, and ZIKV PR-immune animals presented with a moderate disease phenotype, showing a faster recovery compared to flavivirus-naïve, YFV-infected animals (Figure 2a,b). DENV-2 NGC-, DENV-2 P8-, and ZIKV-immune animals had significantly lower YFV viremia compared to flavivirus-naïve animals (*p* < 0.0001) (Figure 2c,d). Upon YFV BR infection, flavivirus-naïve animals had higher viral loads in the kidney and spleen at 2 DPI (Supplementary Figure S6), which reached statistical significance by 4 DPI, compared to flavivirus-immune animals (Figure 2e,f). Except for one DENV-2 NGC-immune kidney being YFV-positive at 4 DPI, all other tissues from immune animals were negative at both timepoints. Interestingly, we could not detect YFV in any of the livers (Figure 2g).

**Figure 3:**
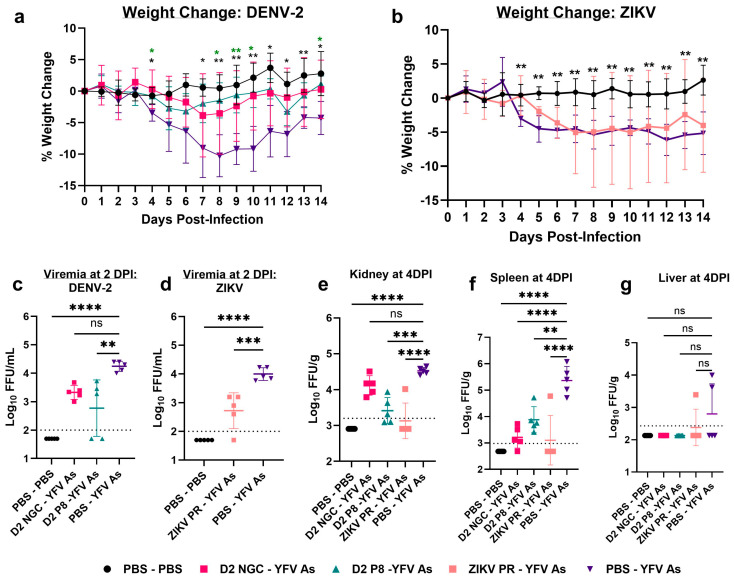
Percentage change in weight and viremia and viral loads in flavivirus- naïve and flavivirus-immune mice, post-YFV Asibi challenge. Seven-to-eight-week-old A129 male mice were first inoculated with PBS or inoculated with DENV-2 NGC (pink), DENV-2 P8 (dark green), or ZIKV PR (peach), and allowed to develop immunity for 7–8 weeks. Mice were then challenged with 10^5^ FFU YFV Asibi and observed for morbidity and mortality, and viremia was measured at 2 DPI. The figure shows the percentage weight change in DENV-2 NGC-, DENV-2 P8- (**a**), and ZIKV-immune (**b**) mice, compared to flavivirus-naïve mice post-YFV Asibi infection, respectively. Viremia levels at 2 DPI in DENV-2 NGC-, DENV-2 P8- (**c**) and ZIKV-immune (**d**) mice, post-YFV Asibi infection, respectively. In a separate study, PBS or YFV Asibi-challenged mice were sacrificed to collect kidney (**e**), spleen (**f**), and liver (**g**) at 4 DPI to determine viral loads. For weight-change graphs, symbols represent the mean, and error bars represent the standard deviation. For viral load graphs, symbols represent individual subjects, midlines represent the mean, and error bars represent the standard deviation. Samples with no detectable YFV are represented as half the lower limit of detection. Weight change within PBS-YFV Asibi group analyzed by two-way ANOVA and repeated measurements by Dunnet’s multiple comparisons test. Log_10_ transformed viremia levels are compared to PBS-YFV BR by one-way ANOVA with Tukey’s multiple comparison test. The *p*-values are as follows: ns: *p* > 0.05; * *p* < 0.05, ** *p* < 0.01, *** *p* < 0.001, and **** *p* < 0.0001. Dotted line represents the limit of detection, and value below is represented as ½ of limit of detection.

Upon infection with YFV Asibi, DENV-2 NGC-, and DENV-2 P8-immune animals were moderately protected against weight loss compared to flavivirus-naïve animals (Figure 3a). Although protection from viremia was not as robust for YFV Asibi as it was for YFV BR, a significant difference was observed between DENV-2 P8-immune and flavivirus-naïve animals, but not for the DENV-2 NGC group (Figure 3c). Interestingly, ZIKV PR-immune animals were not significantly protected against weight loss (Figure 3b); however, a detailed investigation of individual mice from the immune group showed three distinct phenotypes: responders (completely protected against weight loss), non-responders (no protection against weight loss), and enhanced disease (lost more weight compared to the flavivirus-naïve group) (Supplementary Figure S7). Irrespective of the spectrum of the disease, all ZIKV-immune mice had significantly lower YFV viremia, compared to the flavivirus-naïve group (Figure 3d).

At 2 DPI, YFV was detected in the kidneys of most animals from the flavivirus-naïve and flavivirus-immune groups; however, the virus was not detectable in spleens, with the exception of one ZIKV-immune animal (Supplementary Figure S7). At 4 DPI, viral loads were significantly reduced in the kidneys of DENV-2 P8- and ZIKV-immune animals, but not in DENV-2 NGC-immune animals, compared to flavivirus-naïve controls (Figure 3e). This finding was consistent with the viremia levels of the DENV-2 NGC group, suggesting that DENV-2 NGC immunity may not have a major effect on YFV Asibi viremia and viral dissemination to certain organs like the kidneys. However, in spleens, the viral load was significantly lower in DENV-2 NGC-, DENV-2 P8-, and ZIKV-immune groups, compared to flavivirus-naïve groups (Figure 3f). Interestingly, all but one of the ZIKV-immune mice had no detectable virus in spleen (Figure 3f). Viral loads in liver were generally low in all groups (Figure 3g).

### 3.2. CD4^+^ T Cells in DENV-2/ZIKV-Immune Mice Play Minimal Roles in Cross-Protection against YFV Infection

**Figure 4:**
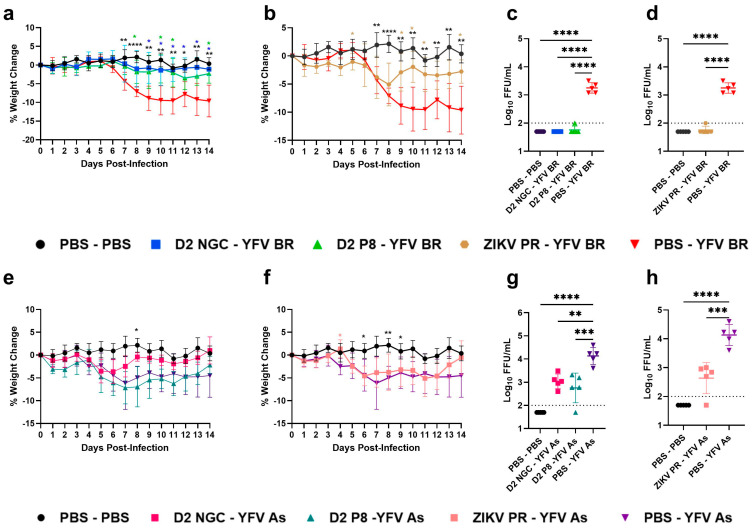
Percentage weight change and viremia levels in CD4+ T cell-depleted, YFV BR- or YFV Asibi-infected mice with varying flavivirus immunity profiles. Seven-to-eight-week-old A129 male mice are first inoculated with PBS or inoculated with DENV-2 NGC, DENV-2 P8, or ZIKV PR, and allowed to develop immunity for 7–8 weeks. Mice were depleted for CD4^+^ T cells prior to challenge with 10^5^ FFU YFV BR or Asibi and observed for morbidity and mortality up to 14 DPI, and viremia was measured at 2 DPI. The figure shows percentage weight change in DENV-2 NGC-, DENV-2 P8- (**a**,**e**), and ZIKV-immune (**b**,**f**) mice compared to flavivirus-naïve mice post-YFV BR or YFV Asibi infection, respectively. Viremia levels at 2 DPI in DENV-2 NGC-, DENV- P8- (**c**,**g**) and ZIKV-immune (**d**,**h**) mice post-YFV BR or Asibi infection, respectively. (**a**–**d**) represent results post-YFV BR challenge and (**e**–**h**) represent results post-YFV Asibi challenge. For weight-change graphs, symbols represent the mean, and error bars represent the standard deviation. For viral load graphs, symbols represent individual subjects, midlines represent the mean, and error bars represent the standard deviation. Weight change in PBS-YFV BR and PBS-YFV Asibi groups analyzed by two-way ANOVA, repeated measurements by Dunnet’s multiple comparisons test. Log_10_ transformed viremia levels are compared to PBS-YFV BR or PBS-YFV Asibi by one-way ANOVA, and Tukey’s multiple comparison test. The *p*-values are as follows: ns: *p* > 0.05; * *p* < 0.05, ** *p* < 0.01, *** *p* < 0.001, and **** *p* < 0.0001. Dotted line represents the limit of detection, and value below is represented as ½ of limit of detection.

To determine the contribution of CD4^+^ T cells in restricting YFV replication in DENV-2- and ZIKV-immune mice, we utilized a CD4^+^ T cell depletion approach. Age-matched A129 males were sham-infected or DENV-2 NGC-, DENV-2 P8-, or ZIKV PR-infected and allowed to develop immunity for 7–8 weeks, similar to our previous design. On days 3 and 1 before the YFV challenge, mice were depleted of CD4^+^ T cells using an anti-CD4 antibody (clone GK1.5) at a dose of 100 µg at each timepoint. Doses and time points were selected based on pilot experiments wherein successful depletion was confirmed on day 3 after the first injection and on day 21, to ensure that cells remained depleted throughout the study. We observed that, despite CD4^+^ T cell depletion, DENV-2- and ZIKV-immune mice were significantly protected against weight loss (Figure 4a,b) caused by YFV BR infection, and had significantly lower YFV viremia (non-detectable in almost all mice) (Figure 4c,d). Upon YFV Asibi challenge, the weight loss in DENV-2 NGC- and ZIKV-immune mice reflected a trend similar to those of the previous infection groups. (Figure 4e,f). Interestingly, DENV-2 P8-immune animals that lacked CD4^+^ T cells lost more weight compared to flavivirus-naïve mice, suggesting a minor contribution of CD4^+^ T cells in cross-protection. However, all DENV-2- and ZIKV-immune mice had significantly lower viremia compared to flavivirus-naïve mice, suggesting that CD4^+^ T cells in DENV-2 or ZIKV-immune mice play little to no role in suppressing YFV viremia (Figure 4g,h). The significant reduction in YFV Asibi viremia in DENV-2 NGC-immune mice compared to flavivirus-naïve mice (Figure 4h), a result that was not observed previously in non-depleted animals, also supports this conclusion.

### 3.3. CD8^+^ T Cells in DENV-2/ZIKV-Immune Mice Play Little to No Role in Suppressing YFV Viremia

**Figure 5:**
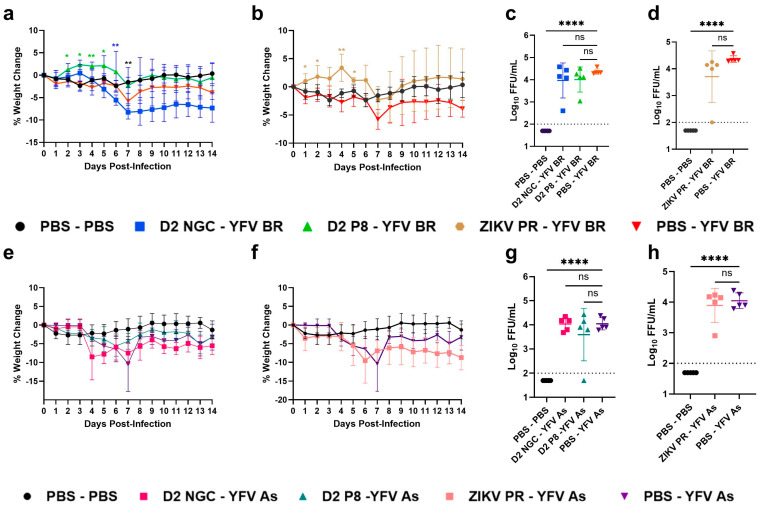
Percentage weight change and viremia in YFV BR- or Asibi-infected mice after adoptive transfer of CD8^+^ T cells from donor mice with varying flavivirus immunity profiles. Seven-to-eight-week-old A129 male mice are first inoculated with PBS or inoculated with DENV-2 NGC, DENV-2 P8, or ZIKV PR, and allowed to develop immunity for 7–8 weeks. Pooled spleens were utilized for CD8^+^ T cell isolation and adoptive transfer to naïve recipient mice one day prior to challenge with 10^5^ FFU YFV BR or Asibi. Mice were observed for morbidity and mortality up to 14 DPI, and viremia was measured at 2 DPI. The figure shows percentage weight change in DENV-2 NGC-, DENV-2 P8- (**a**,**e**) and ZIKV-immune (**b**,**f**) mice, compared to flavivirus-naïve mice, post-YFV BR or Asibi infection, respectively. Viremia levels at 2 DPI in DENV-2 NGC-, DENV- P8- (**c**,**g**) and ZIKV-immune (**d**,**h**) mice post-YFV BR or Asibi infection, respectively. (**a**–**d**) represent results post-YFV BR challenge and (**e**–**h**) represent results post-YFV Asibi challenge. For weight-change graphs, symbols represent the mean, and error bars represent the standard deviation. For viral load graphs, symbols represent individual subjects, midlines represent the mean, and error bars represent the standard deviation. Weight change in PBS-YFV BR and PBS-YFV Asibi groups analyzed by two-way ANOVA, repeated measurements with Dunnet’s multiple comparisons test. Log_10_ transformed viremia levels are compared to PBS-YFV BR or PBS-YFV Asibi by one-way ANOVA, and Tukey’s multiple comparison test. The *p*-values are as follows: ns: *p* > 0.05; * *p* < 0.05, ** *p* < 0.01, and **** *p* < 0.0001. Dotted line represents the limit of detection, and value below is represented as ½ of limit of detection.

To determine the contribution of CD8**^+^** T cells to cross-protection, we adoptively transferred CD8^+^ T cells from sham-infected or DENV-2- or ZIKV-infected donor mice that were allowed to develop immunity for 7–8 weeks (similar duration to previous studies) to naïve recipient mice, and challenged them one day later with YFV BR or Asibi. Before adoptive transfer, the purity of the CD8**^+^** T cell population was confirmed to be >95% by flow cytometry, and 10^6^ cells per mouse were transferred. All other parameters were identical to the previous approach. We found that transferring CD8**^+^** T cells from DENV-2 NGC-, DENV-2 P8- or ZIKV-immune mice generally did not protect against weight loss due to YFV BR or Asibi infection (Figure 5a,b,e,f). In fact, DENV-2 NGC-derived CD8 recipients lost more weight compared to flavivirus-naïve mice upon YFV BR and Asibi infection, suggesting a possible exacerbating role of CD8**^+^** T cells. No significant difference in viremia between animals receiving CD8^+^ cells from immune versus naïve mice was observed (Figure 5c,d,g,h). These results together show that adoptively transferring one million CD8**^+^** T cells from donor mice did not suppress YFV disease or viremia in A129 mice.

### 3.4. Antibodies Contribute to Cross-Protection in Sequential DENV-2/ZIKV and YFV Infections

**Figure 6:**
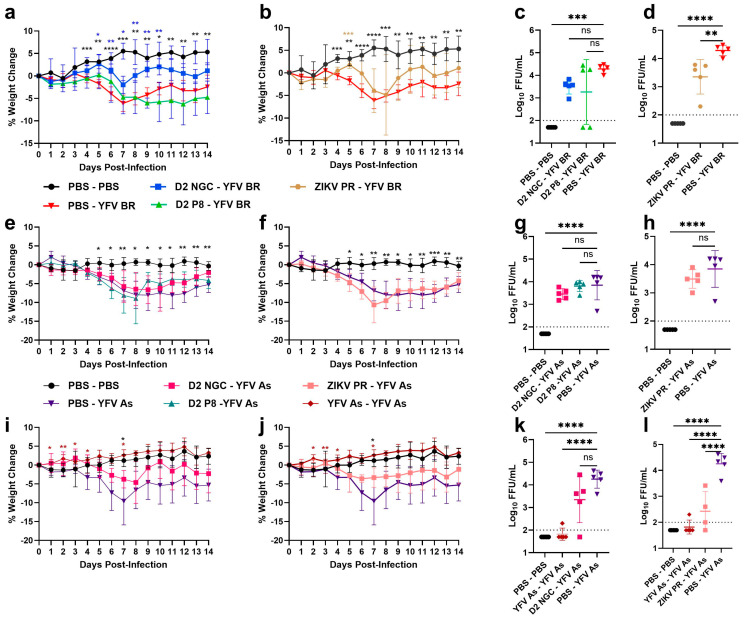
Weight change and viremia levels in YFV BR- or Asibi-infected mice with sera passively transferred from donor mice with varying flavivirus immunity profiles. Seven-to-eight-week-old A129 male mice were first inoculated with PBS or inoculated with DENV-2 NGC (pink), DENV-2 P8 (dark green), or ZIKV PR (peach), and allowed to develop immunity for 7–8 weeks. Pooled sera were collected and passively transferred to naïve recipient mice (100 µL per mouse) one day prior to challenge with 10^5^ FFU YFV BR or Asibi. Mice were observed for morbidity and mortality up to 14 DPI, and viremia was measured at 2 DPI. The figure shows percentage weight change in DENV-2 NGC-, DENV-2 P8- (**a**), and ZIKV-immune (**b**) mice compared to flavivirus-naïve mice post-YFV BR infection. Viremia levels at 2 DPI in DENV-2 NGC-, DENV- P8- (**c**) and ZIKV-immune (**d**) mice post-YFV BR infection. Percentage weight change in DENV-2 NGC-, DENV-2 P8- (**e**), and ZIKV-immune (**f**) mice, compared to flavivirus-naïve mice post-YFV Asibi infection. Viremia levels at 2 DPI in DENV-2 NGC-, DENV- P8- (**g**) and ZIKV-immune (**h**) mice post-YFV Asibi infection. YFV Asibi challenge was repeated to include a positive control that received YFV Asibi-immune sera (red), with an increase in the volume of sera transferred in all the groups to 150 µL per mouse (**i**–**l**). Weights (7i, 7j) and viremia (7k, 7l) were measured as previously described. For weight-change graphs, symbols represent the mean, and error bars represent the standard deviation. For viral load graphs, symbols represent individual subjects, midlines represent the mean, and error bars represent the standard deviation. Weight change was compared to PBS-YFV BR or PBS-YFV Asibi group by two-way ANOVA, and repeated measurements with Dunnet’s multiple comparisons test. Log_10_ transformed viremia levels are compared to PBS-YFV BR or PBS-YFV Asibi by one-way ANOVA, and Tukey’s multiple comparison test. The *p*-values are as follows: ns: *p* > 0.05; * *p* < 0.05, ** *p* < 0.01, *** *p* < 0.001, and **** *p* < 0.0001. Dotted line represents the limit of detection, and value below is represented as ½ of limit of detection.

Typically, neutralizing antibodies are considered the primary correlate of protection against homologous flavivirus infection. To determine if antibodies contributed to cross-protection, we passively transferred 100 µL of pooled sera from sham-infected or DENV-2- or ZIKV-infected donor mice to recipient mice and challenged with YFV BR or Asibi. Upon YFV BR challenge, animals receiving DENV-2 NGC-immune serum were significantly protected against weight loss compared to those receiving flavivirus-naïve serum (Figure 6a). Although not significant, a general trend of lower viremia was observed in the former group (Figure 6c). Mice receiving DENV-2 P8-immune serum showed no overall protection against weight loss, although two of the five animals had non-detectable viremia (Figure 6a,c). Animals receiving ZIKV-immune serum were not protected against weight change, and showed 20% lethality (1/5), but had significantly lower viremia compared to mice that received flavivirus-naïve serum (Figure 6b,d).

Upon YFV-Asibi challenge, the immune serum groups were not protected against weight change or viremia (Figure 6e–h). We repeated the study by transferring 150 µL of donor sera and included a positive control group that received comparable levels of YFV Asibi-immune serum. Due to limited availability of animals, we only included one DENV strain (DENV-2 NGC) in these studies. As expected, the positive control group was completely protected against weight loss, disease, and viremia. Mouse groups that received DENV-NGC- (80% survival) or ZIKV-immune (100% survival) serum had mild, but not statistically significant, protection against weight loss compared to those that received flavivirus-naïve serum (60% survival) (Figure 6i,j). DENV-2 NGC-transferred groups had viremia that, although not statistically significant, trended lower compared to the naïve serum transfer group (Figure 6k). ZIKV-PR-transferred animals had significantly lower viremia compared to the naïve group (Figure 6l).

### 3.5. Serum Chemistries of YFV Asibi-Infected Mice with Varying Flavivirus Immune Profiles Generally Demonstrate No Significant Differences

**Figure 7:**
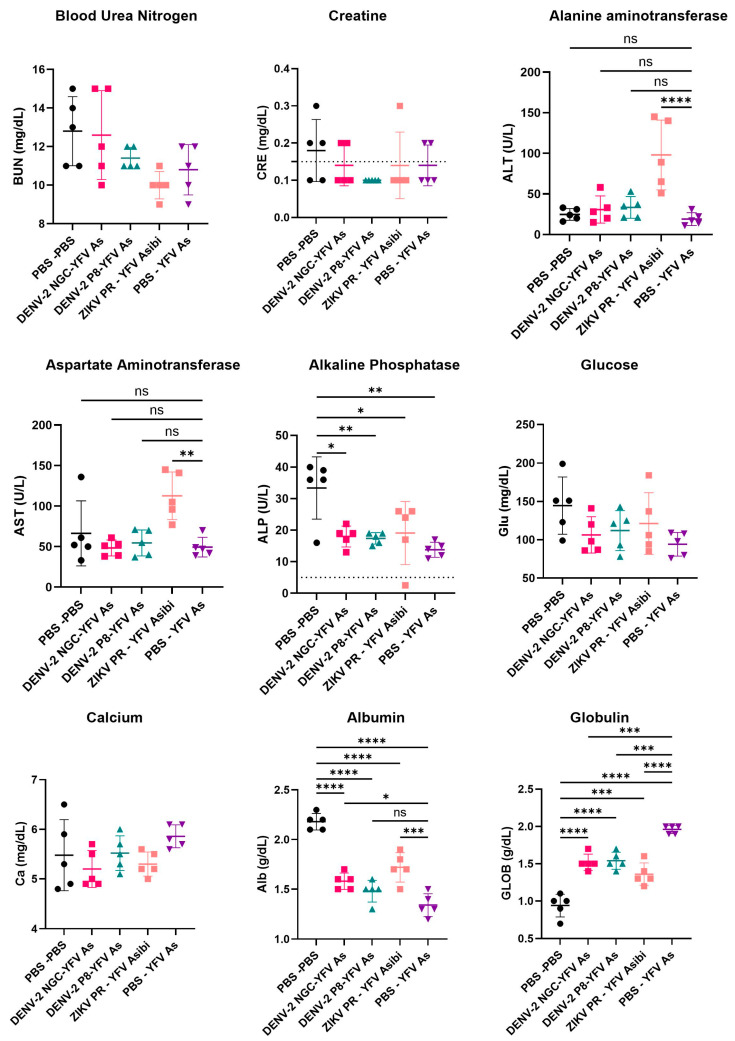
**Serum chemistries of YFV Asibi-infected mice at 4 DPI with varying flavivirus immunity**. Mice primarily inoculated with either PBS, DENV-2 NGC, DENV-2 P8, or ZIKV PR YFV were allowed to develop immunity for 7–8 weeks followed by a mock or a YFV Asibi challenge. The sera at 4 DPI from Asibi-infected mice were utilized to determine levels of blood urea nitrogen (BUN), creatine (CRE), alanine aminotransferase (ALT), aspartate aminotransferase (AST), alkaline phosphatase (ALP), glucose (GLU), calcium (Ca), albumin (Alb), and globulin (GLOB). For all graphs, symbols represent individual subjects, midlines represent the mean, and error bars represent the standard deviations. Data from each group was combined and analyzed by one-way ANOVA with Dunnett’s multiple comparison test. ns: *p* > 0.05, * *p* < 0.05, ** *p* < 0.01, *** *p* < 0.001, **** *p* < 0.0001.

Sera collected at 4 DPI from YFV Asibi-infected mice previously inoculated with either PBS, DENV-2 NGC, DENV-2 P8, or ZIKV PR and allowed to build immunity for 7–8 weeks immunity (Section 3.2) were utilized to measure serum chemistries by Vetscan. No significant difference in levels of blood urea nitrogen (BUN), creatine (CRE), glucose, or calcium were observed between the different groups (Figure 7). All groups (flavivirus-naïve and flavivirus-immune) had significantly lower levels of alkaline phosphatase and albumin and significantly higher levels of globulin compared to the PBS mock. Interestingly, only ZIKV-immune mice had elevated levels of the liver enzymes alanine aminotransferase (ALT) and aspartate aminotransferase (AST), suggesting a higher level of damage to the liver compared to other groups.

## 4. Discussion

Experimental studies to investigate a pathogen often focus only on that specific pathogen, discounting the influence of immunity from related pathogens in endemic regions or in an outbreak setting. In the context of flaviviruses, numerous human cohort studies focus on understanding the immune response and the role of pre-existing immunity from primary infection in the course of disease during secondary infection, a phenomenon that has particularly been important in DENV infections due to its association with antibody-dependent enhancement (ADE) [32,33,34]. Several studies seeking to determine the role of DENV immunity in ZIKV infection and vice versa have been reported since the massive ZIKV outbreak starting in 2015 in South America, a region that is hyper-endemic for DENV [34,35,36,37]. It has been shown, particularly through experimental studies, that prior immunity to one flavivirus influences the secondary infection [38,39,40,41].

Here, we attempted to address the discrepancy between locations of urban YFV activity and the availability of naïve human amplification hosts, as well as *Ae. aegypti* mosquitoes, a long-standing enigma in arbovirology. Considering the overlapping geographical distribution of DENV and ZIKV, along with the complete absence or inconsistent urban occurrence of YF, we were prompted to explore the potential influence of prior immunity to DENV or ZIKV on YFV infection, viremia, and disease progression.

A129 mice, which are susceptible to YFV infection and viremia, were utilized as the primary animal model, first infected with either DENV-2 or ZIKV to induce immunity over a period of 7–8 weeks. Subsequently, they were challenged with either African or Brazilian strains of YFV. Our findings revealed that mice previously exposed to DENV or ZIKV exhibited significantly reduced YFV viremia and displayed partial protection against disease progression. Notably, when challenged with YFV BR, there was a remarkable suppression of viremia and viral load in the organs of all immune animals. While a similar suppression was observed upon challenge with YFV Asibi, which is generally more virulent, the disparity was less pronounced, compared to the YFV BR challenge. These results collectively suggest the following: (1) Immunity acquired from prior DENV and ZIKV infections contributes significantly to attenuating YFV viremia, but may or may not influence the disease. (2) Strain-specific YFV variation exists in the inhibition by prior flavivirus immunity, with the Brazilian YFV strain demonstrating greater susceptibility. (3) Furthermore, differences were noted between groups with immunity to two different DENV strains (sylvatic and endemic), indicating that strains circulating in a particular region might also influence the viral transmission dynamics of a related flavivirus.

Next, we investigated the roles of CD4^+^ T cells, CD8^+^ T cells, and antibodies in sequential DENV/ZIKV and YFV infections, using depletion and adoptive transfer approaches. Our results indicate that CD4^+^ and CD8^+^ T cells contribute minimally to suppressing YFV viremia. Interestingly, passive transfer of sera demonstrated partial protection as indicated by significantly lower viremia in ZIKV-immune mice, and viremia levels generally trending lower in DENV-immune mice. The protection was enhanced upon increasing the volume of sera transferred, further supporting the role of serum antibodies in cross-protection.

In our studies, we used two different YFV strains representing historic African strains and contemporary Brazilian strains and found a strain-specific difference in the response to prior flavivirus immunity. We also used two different DENV-2 strains for primary infection and found a difference in their effect on YFV challenge, underscoring the complexity of cross-protection. Additionally, we allowed mice to develop an immune response after the primary infection for approximately two months in all of our studies, mimicking a convalescent phase response. Moreover, these results corroborate with our findings in NHPs, where we specifically investigated the impact of prior flavivirus immunity on mosquito infection during YFV viremia, emphasizing the potential role of heterologous flavivirus immunity on human amplification [31].

We measured serum chemistries at 4 DPI and generally found no striking differences between flavivirus-naïve and flavivirus-immune groups. Albumin levels were lower in all groups compared to the PBS mock group, suggesting liver or kidney damage; however, the levels were higher in flavivirus-immune animals compared to flavivirus-naïve animals. Measuring serum chemistries at a later timepoint after challenge, or in NHPs that are challenged with a virulent YFV strain, may provide better information on the impact of flavivirus immunity on YF disease course. Interestingly, we observed an exacerbating effect of ZIKV immunity in some YFV-infected animals, particularly reflected by higher ALT and AST levels in ZIKV-immune mice compared to ZIKV-naïve mice. In a typical group of five mice, one ZIKV-immune mouse consistently showed a higher drop in weight, higher lethality, or higher viral loads. The elevations in liver enzymes are particularly intriguing, suggesting a possibility that in a subset of the ZIKV-immune population, YF disease may be exacerbated, potentially by ADE, a phenomenon that has been established in sequential DENV infections. While similar observations in ZIKV-immune NHPs were noted in our previous studies [31], more experiments to specifically investigate this phenomenon are needed.

There are a few limitations associated with our studies: (1) Since WT mice are resistant to YFV infection, we utilized an immune-compromised mouse model. Although it is not uncommon to use A129 mice to study flaviviruses and adaptive immune responses [13,42], we recognize that the lack of interferon α/β receptors may have some downstream effects. Additional studies, perhaps utilizing nonhuman primates or rodent-adapted viral strains, would be useful to confirm these results in the future. (2) We utilized two different strains of DENV-2; it would be beneficial to investigate the effect of other serotypes. (3) Our results demonstrate that neither CD4^+^ nor CD8^+^ T cells play a major role in cross-protection. However, future detailed studies investigating specific immune responses are needed.

In summary, we demonstrated a substantial effect of flavivirus immunity on suppressing YFV viremia in A129 mice, which appears to be primarily provided by the humoral arm of immunity. It would also be interesting to know if prior immunity to DENV/ZIKV can skew YF disease severity and pathogenesis in humans. The roles of other DENV serotypes, and other flaviviruses, like JEV, that are widespread in Asia, should also be investigated. Furthermore, with a larger population receiving vaccinations like JEV, or more recently DENV, the impact of non-YFV flavivirus vaccination on YFV transmission dynamics also merits further evaluation.

## Data Availability

Data are available upon request.

## Supplementary Materials

The following supporting information can be downloaded at: https://www.mdpi.com/article/10.3390/v16060836/s1.
