# Supplementary material for: Mechanisms of Flavivirus Cross-Protection against Yellow Fever in a Mouse Model
Source: Viruses. 2024 May 24;16(6):836. doi: 10.3390/v16060836 (PMC11209131; doi:10.3390/v16060836)
Supplement: Supplementary file 1 [file viruses-16-00836-s001.zip › viruses-2981383-supplementary.pdf]

Supplementary Information

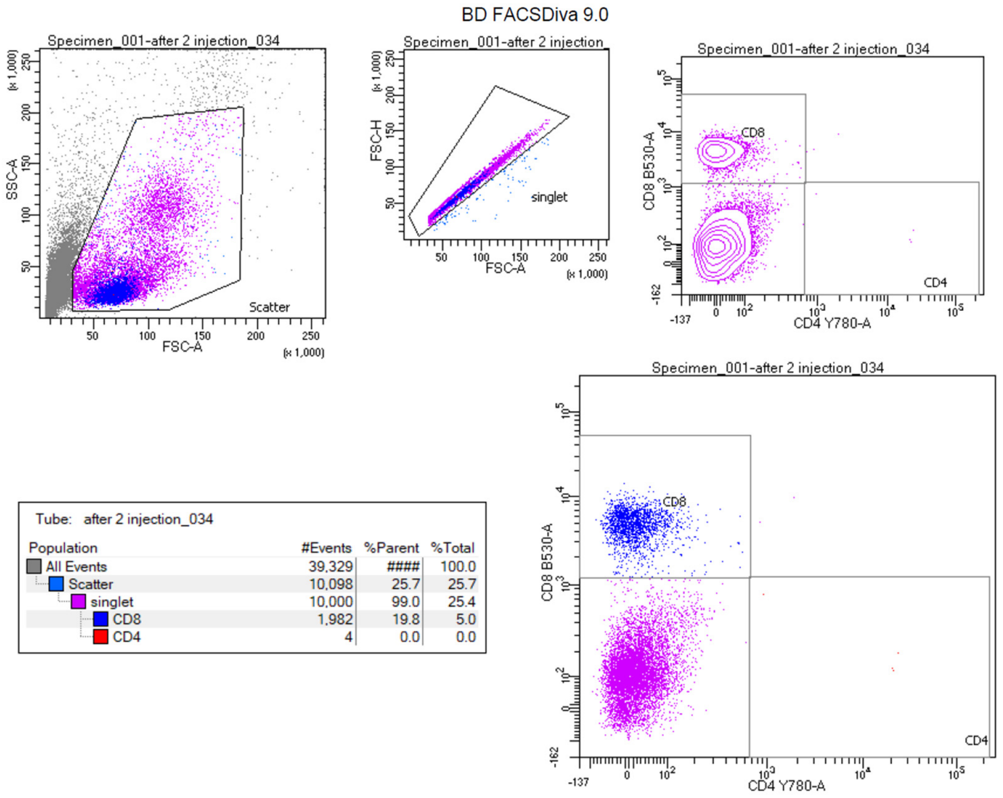

Population	#Events	%Parent	%Total
All Events	39,329	####	100.0
Scatter	10,098	25.7	25.7
singlet	10,000	99.0	25.4
CD8	1,982	19.8	5.0
CD4	4	0.0	0.0

Supplementary Figure S1: Successful depletion of $CD4^{+}$ T cells after two injections of 100µg on days 3 and 1 before YFV infection.

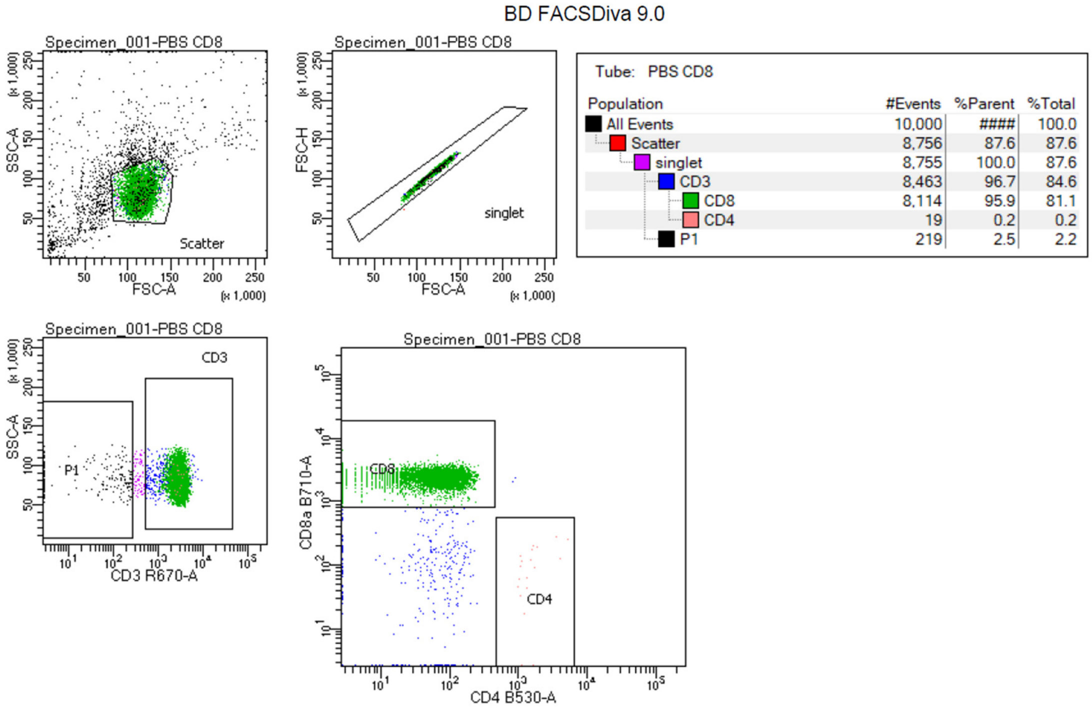

Supplementary Figure S2: CD8+ T cells isolated from spleens of PBS inoculated mice using Miltenyi CD8a^{+} T cell isolation kit for mouse. Purity confirmed to be 95.9%.

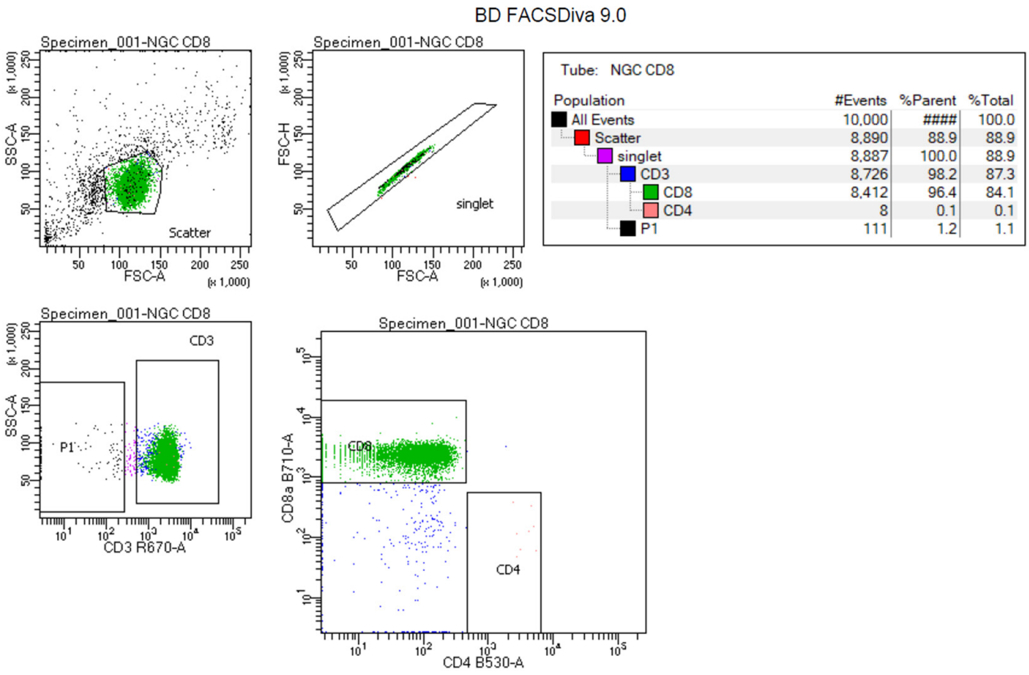

Supplementary Figure S3: CD8+ T cells isolated from spleens of DENV-2 NGC-inoculated mice using Miltenyi CD8a^{+} T cell isolation kit for mouse. Purity confirmed to be 96.4%.

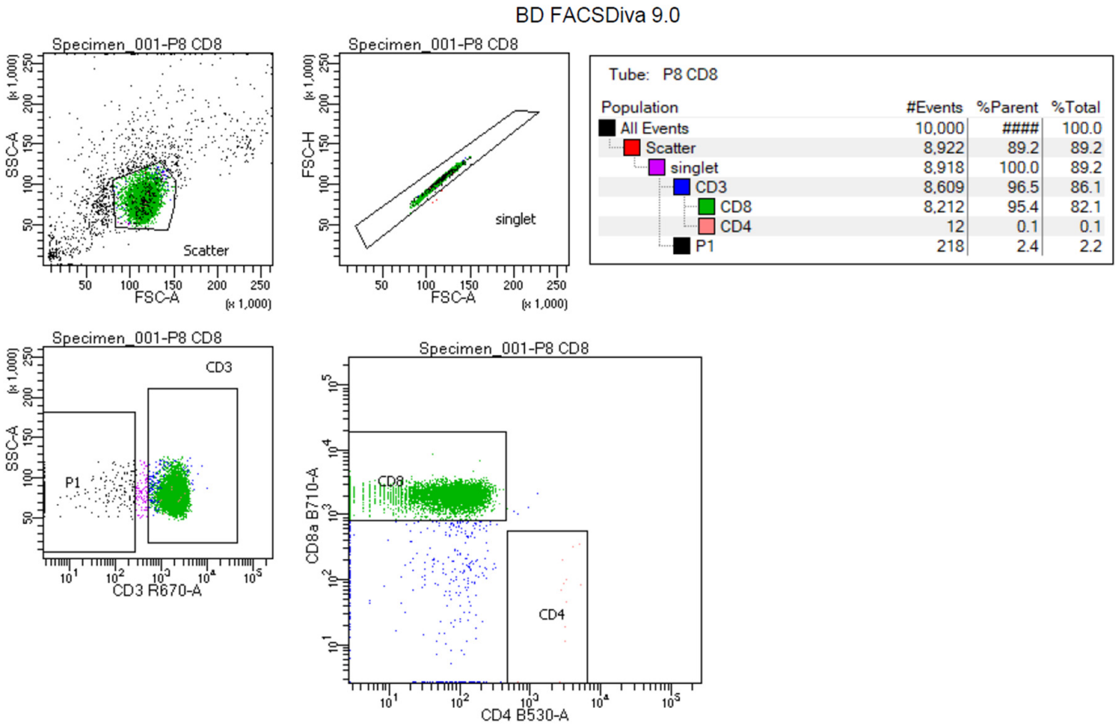

Supplementary Figure S4: CD8+ T cells isolated from spleens of DENV-2 P8-inoculated mice using Miltenyi CD8a^{+} T cell isolation kit for mouse. Purity confirmed to be 95.4%.

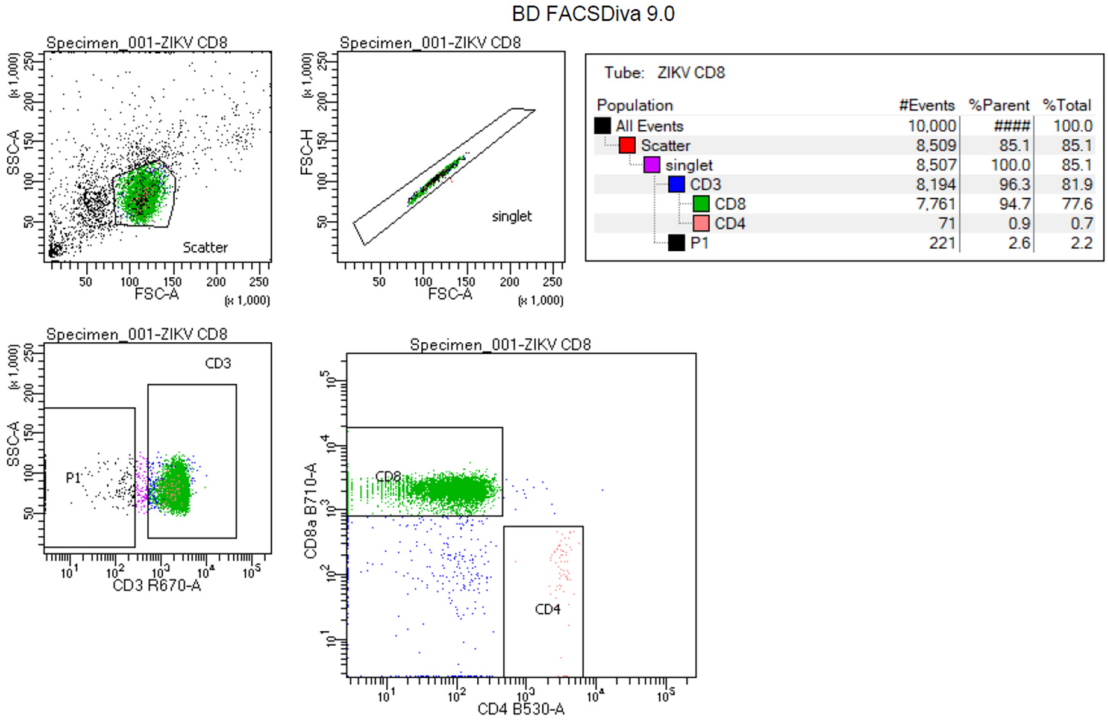

Supplementary Figure S5: CD8+ T cells isolated from spleens of ZIKV-PR-inoculated mice using Miltenyi CD8a^{+} T cell isolation kit for mouse. Purity confirmed to be 94.7%.

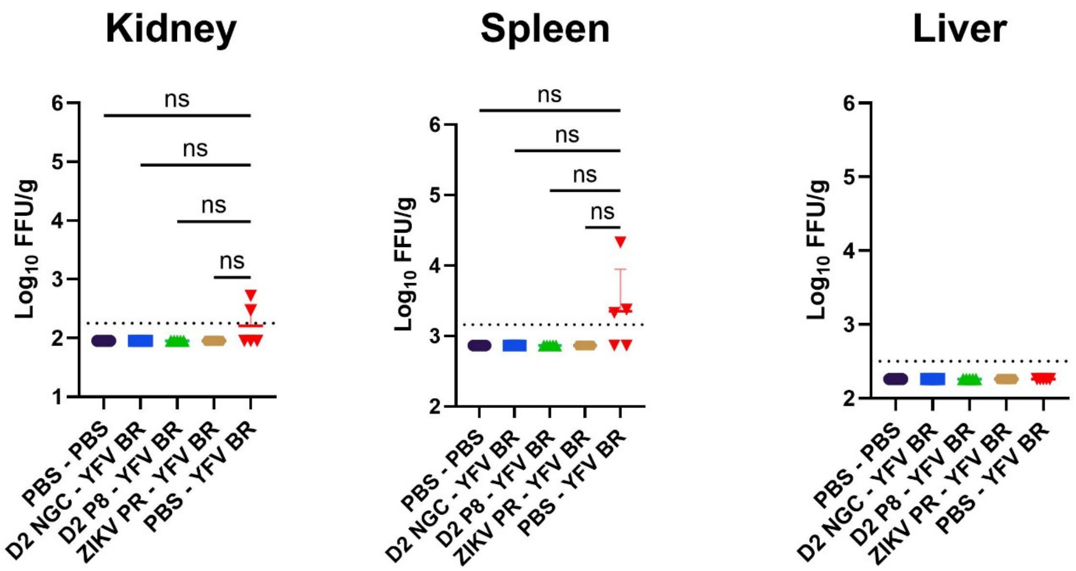

Supplementary Figure S6: Viral loads in flavivirus-naive and -immune mice at 2 DPI, post YFV BR challenge. Seven-eight-week-old A129 male mice were first inoculated with PBS or DENV-2 NGC, DENV-2 P8, or ZIKV PR, and allowed to develop immunity for 7-8 weeks. Mice were then challenged with 10^5 FFU YFV BR. Mice were sacrificed to collect kidney, spleen, and liver at 4 DPI to determine viral loads.

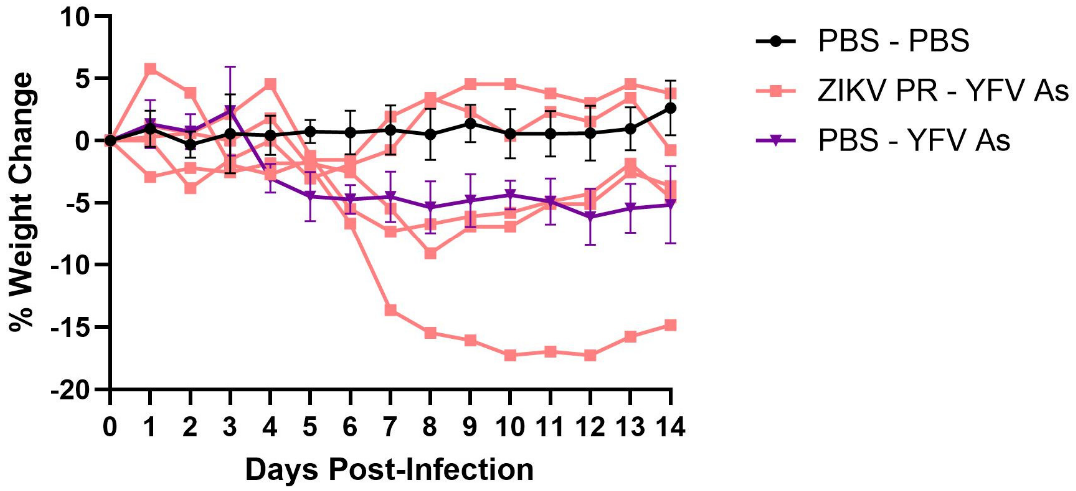

Supplementary Figure S7: Fifteen-sixteen-week-old, A129 males primarily inoculated with PBS or ZIKV PR was challenged with YFV Asibi and observed for weight change. ZIKV-immune mice show complete protection in 2/5 mice, no protection in 2/5 mice, and worse disease in 1/5 mice.

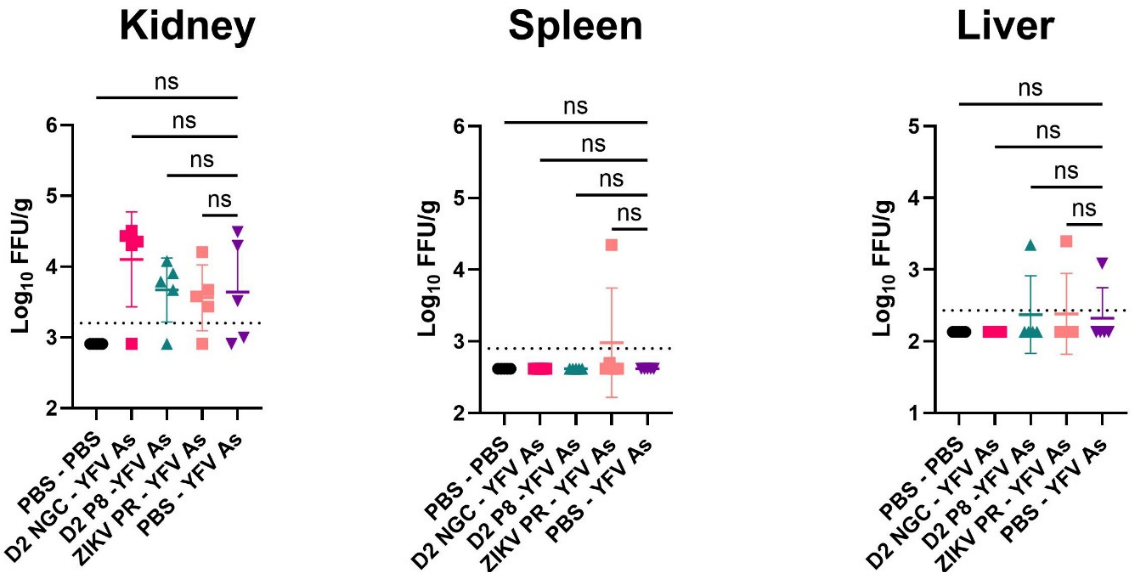

Supplementary Figure S8: Viral loads in flavivirus-naive and -immune mice at 2 DPI, post YFV Asibi challenge. Seven-eight-week-old A129 male mice were first inoculated with PBS or DENV-2 NGC, DENV-2 P8, or ZIKV PR, and allowed to develop immunity for 7-8 weeks. Mice were then challenged with 10^5 FFU YFV Asibi. Mice were sacrificed to collect kidney, spleen, and liver at 4 DPI to determine viral loads.
